# Delivering online alternatives to the anatomy laboratory: Early experience during the COVID‐19 pandemic

**DOI:** 10.1002/ca.23722

**Published:** 2021-02-08

**Authors:** William Flynn, Naveen Kumar, Russell Donovan, Melissa Jones, Paula Vickerton

**Affiliations:** ^1^ Department of Anatomy Barts and the London School of Medicine and Dentistry, Queen Mary University of London UK

**Keywords:** anatomy, embryology, medical education, medicine, surgery

## Abstract

Social distancing measures due to the COVID‐19 pandemic will make anatomy dissecting room practicals difficult, if not impossible to run at some institutions in the upcoming academic year. The learning community that exists within physical anatomy practicals needs to be moved online. Virtual replacement of visuo‐spatial and social elements of learning anatomy pose particular challenges to educators. Our department has trialed Blackboard Collaborate, an online communication platform in conjunction with Visible Body, a 3D anatomical modeling program. We have delivered 266 hr of synchronous small group teaching to medical and physician associate students. We describe this approach and discuss the relevance of distance learning pedagogy to the design of new online anatomy teaching and development of online learning communities.

## 1 INTRODUCTION

On March 23, 2020, the UK government implemented a national lockdown due to the COVID‐19 pandemic, which halted all face‐to‐face (F2F) teaching at UK based universities (UK Government, 2020). Faculties raced to develop remote, virtual alternatives to teaching (Evans et al., 2020). Our institution has been preparing online teaching based on the assumption of continued cancellation of large group F2F teaching in the 2020/21 academic year. The utilization of virtual resources in anatomy education has grown significantly over the past two decades (Trelease, 2015). However, until the pandemic, the mainstay of anatomy teaching in the United Kingdom (UK) and Republic of Ireland (ROI) remained the use of cadaveric material in practical classes and dissections (Heylings, 2002; Longhurst et al., 2020). This included our institution, where anatomy education involved lectures, prosection‐based practicals and asynchronous online resources with dissection available in student selected components of the course. While it has been relatively straightforward to replace live lectures by distributing lecture recordings, it is less clear how learning achieved in practical classes and dissections can be replaced online.

The challenges faced by anatomy departments in achieving this have been highlighted recently by Longhurst et al. (2020). They analyzed concerns and adaptations of 14 anatomy departments in the UK and ROI during the COVID‐19 pandemic. The removal of physical practicals evoked concerns amongst educators including reduced visuospatial learning, the loss of student–student and student‐teacher interactions and maintaining student engagement with virtual resources. Most institutions adapted to lockdown by increasing the availability of asynchronous resources. These included virtual cadaveric resources such as Acland's Video Atlas of Human Anatomy (Lippincott Williams & Wilkins, PA) and 3D modeling programs such as Visible Body (Argosy Publishing Inc., MA) and Complete Anatomy (3D4Medical/Elsevier, Dublin, Republic of Ireland). A minority of institutions had implemented synchronous webinars, using Zoom (Zoom Voice Communications Inc., CA), Microsoft Teams (Microsoft, WA) and BigBlueButton (BigBlueButton Inc., CT).

Our department has implemented virtual anatomy practicals for medical students and physician associate students over 2 months during lockdown using “Blackboard Collaborate” (BBC). Its use as a virtual classroom in anatomy education has been demonstrated previously in the literature (Attardi, Barbeau, & Rogers, 2018; Attardi & Rogers, 2015). This platform now offers functions with great interactive and collaborative potential, but their use has not yet been described in anatomy education. Given educators' concerns regarding loss of F2F practicals, we describe our approach to online anatomy education during lockdown, which exploited these functions in an attempt to enhance student–student and student‐teacher interactions and grow a virtual learning community. To distinguish them from their F2F equivalents, we will refer to these online practicals as webinars.

## 2 THEORETICAL BASIS

As pointed out by anatomy teachers (Longhurst et al., 2020), the COVID‐19 pandemic and its aftermath present a unique opportunity for innovation within anatomy education. However, when implementing new learning technologies, there is a risk of supporting existing practice rather than exploiting new pedagogies (Grainger, Liu, & Geertshuis, 2020). There have been calls to ensure evidence‐based pedagogy remains at the forefront of anatomy education innovation (Evans et al., 2020). We can learn from the great amount of theoretical work done in the field of distance learning, to guide the development of online anatomy education.

Learning cannot be separated from the social contexts in which it occurs (Brown, Collins, & Duguid, 1989). In classrooms, interactions between participants can been said to create a community of inquiry where learning is the product of discussion and debate (Lipman, 2003). In the anatomy dissecting room, students agree that interaction with peers and staff is helpful for their learning (Johnson, Palmer, Burton, & Brockhouse, 2013). Learning communities are not only important in the physical world, but are argued to be the most important factor in establishing successful online learning (Palloff & Pratt, 2007) and attempts to develop them are widely advocated in distance learning literature (Fiock, 2020). The concept of a community of inquiry was applied to distance learning courses by academics at the University of Alberta in Canada who published a *Community of Inquiry* (CoI) framework (Garrison, Anderson, & Archer, 2000). This collaborative‐constructivist model aimed to highlight the core components of a community of inquiry, to ensure that they are maintained when moving education from physical to virtual learning environments. It was initially developed for information technology education using asynchronous computer mediated communication (CMC) and has since become widely used in both asynchronous and synchronous online education (Castellanos‐Reyes, 2020).

Garrison (2011) defined a CoI as “a group of individuals who collaboratively engage in purposeful critical discourse and reflection to construct personal meaning and confirm mutual understanding” (p. 15). They describe three key components required to provide a good educational experience within a CoI: social presence, cognitive presence, and teaching presence. Social presence is described as “the ability of participants in the Community of Inquiry to project their personal characteristics into the community, thereby presenting themselves to the other participants as real people” (Garrison et al., 2000, p. 89). It is provided by an environment in which participants can interact comfortably and freely. This can be used to grow a cognitive presence, defined as “the extent to which learners are able to construct and confirm meaning through sustained reflection and discourse in a critical community of inquiry” (Garrison, Anderson, & Archer, 2001, p. 11). In other words, a cognitive presence refers to learning that occurs through discussion between students and teachers. Teaching presence refers to both the design and facilitation of educational experiences to enhance cognitive and social processes (Anderson, Rourke, Garrison, & Archer, 2001). By considering these three presences we can explore how best to develop functioning virtual learning communities in anatomy education.

## 3 MATERIALS AND METHODS

### 3.1 Setting

Our faculty consisted of five full‐time educators: two anatomists and three clinical teaching fellows. Student cohorts taught included 1st year and 2nd year medical undergraduate students, postgraduate medical students and physician associate students. Topics taught during this period mirrored those that would have been addressed during cancelled F2F practicals, and included neuroanatomy, reproductive anatomy, and musculoskeletal anatomy. Our institution provides a systems based, spiral, vertically and horizontally integrated medical curriculum and online teaching was designed to remain in line with this.

### 3.2 Design

Blackboard Collaborate webinars were hosted on the university's online learning environment (Moodle). Students were distributed non‐randomly into existing groups of between eight and 16 students who were already known to each other from previous F2F teaching. Webinars were scheduled to last 90 min, reflecting the time allocated for equivalent F2F practical classes. Many international students are enrolled on our course and scheduling took into account time zone limitations, with students allowed to swap groups for more appropriate timings. Groups were allocated the same teacher for each session to assist continuity. Each webinar centered on a slideshow presentation developed and uploaded by faculty members to the BBC platform in advance. The presentations were constructed using PowerPoint 2013 (Microsoft, WA) and addressed a standardized set of learning outcomes, while allowing for tailoring of content to specific group needs. Webinar slides included some more traditional text and diagrams, but emphasis was placed on incorporating clinical, radiological, and cadaveric images wherever possible.

### 3.3 Communication

Blackboard Collaborate can facilitate a bidirectional flow of written, oral and visual communication. In our webinars, tutors communicated with the students through audio‐visual means. Students were allowed to interact in whichever way they found most comfortable, which included writing or drawing on the slides, typing into an instant messaging chat bar, or by using their microphones. Where background noise became disruptive, students were asked to turn off microphones and turn on again as needed.

### 3.4 Facilitation

**FIGURE 1:**
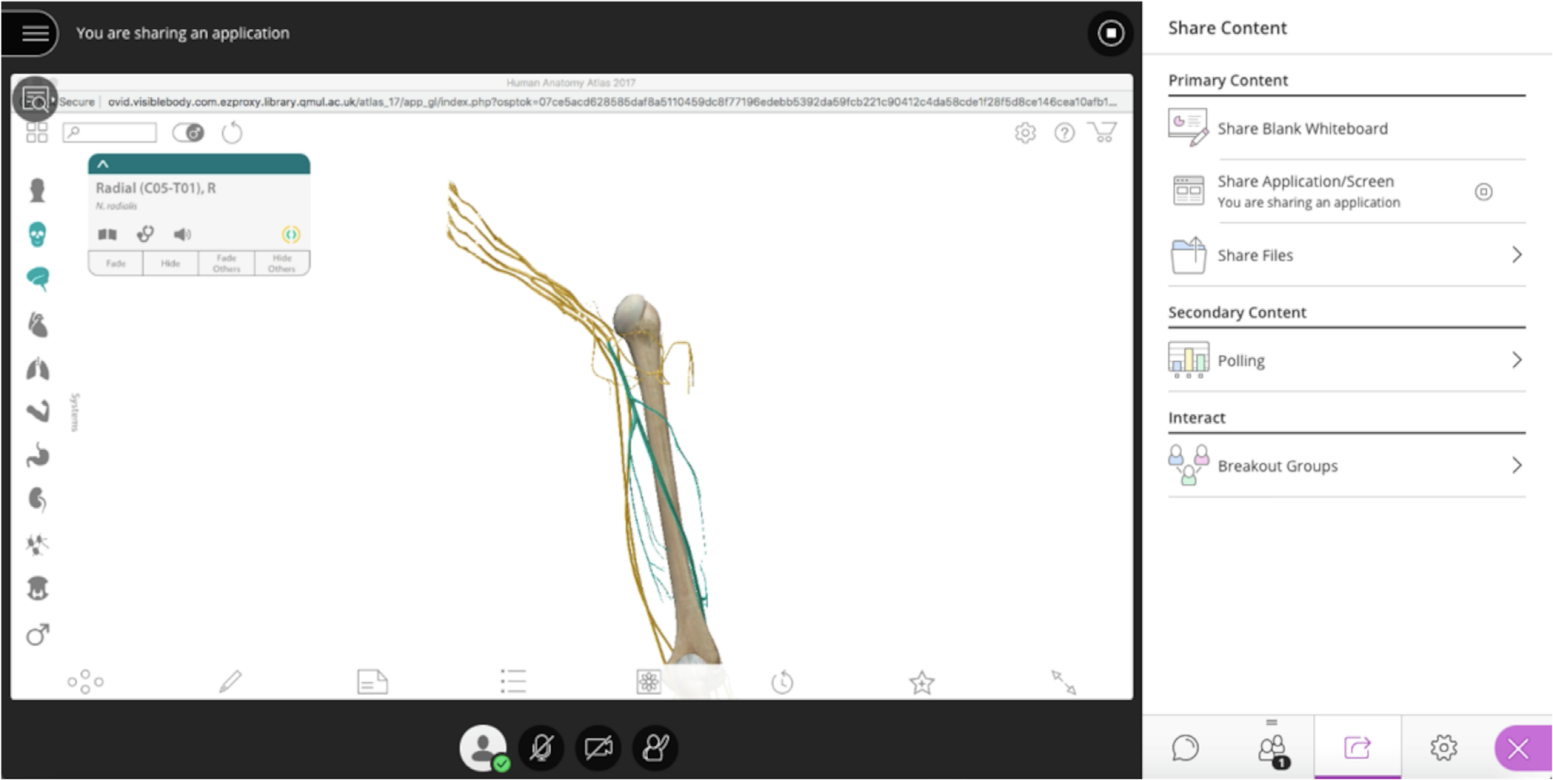
An example of screensharing of Visible Body in BBC. BBC, Blackboard Collaborate [Color figure can be viewed at wileyonlinelibrary.com]

Faculty members guided students through slideshow content, which incorporated demonstrations of other programs through screensharing, and various learning activities. We broadcast Visible Body 3D models (Figure 1) and manipulated them in a similar way to that of physical models in the dissecting room, particularly to demonstrate concepts that are difficult to understand in 2D representations. In BBC sessions, all students in the group were shown the teacher's model on screen, and the models were also freely available to students to access individually at any time.

### 3.5 Interaction

**FIGURE 2:**
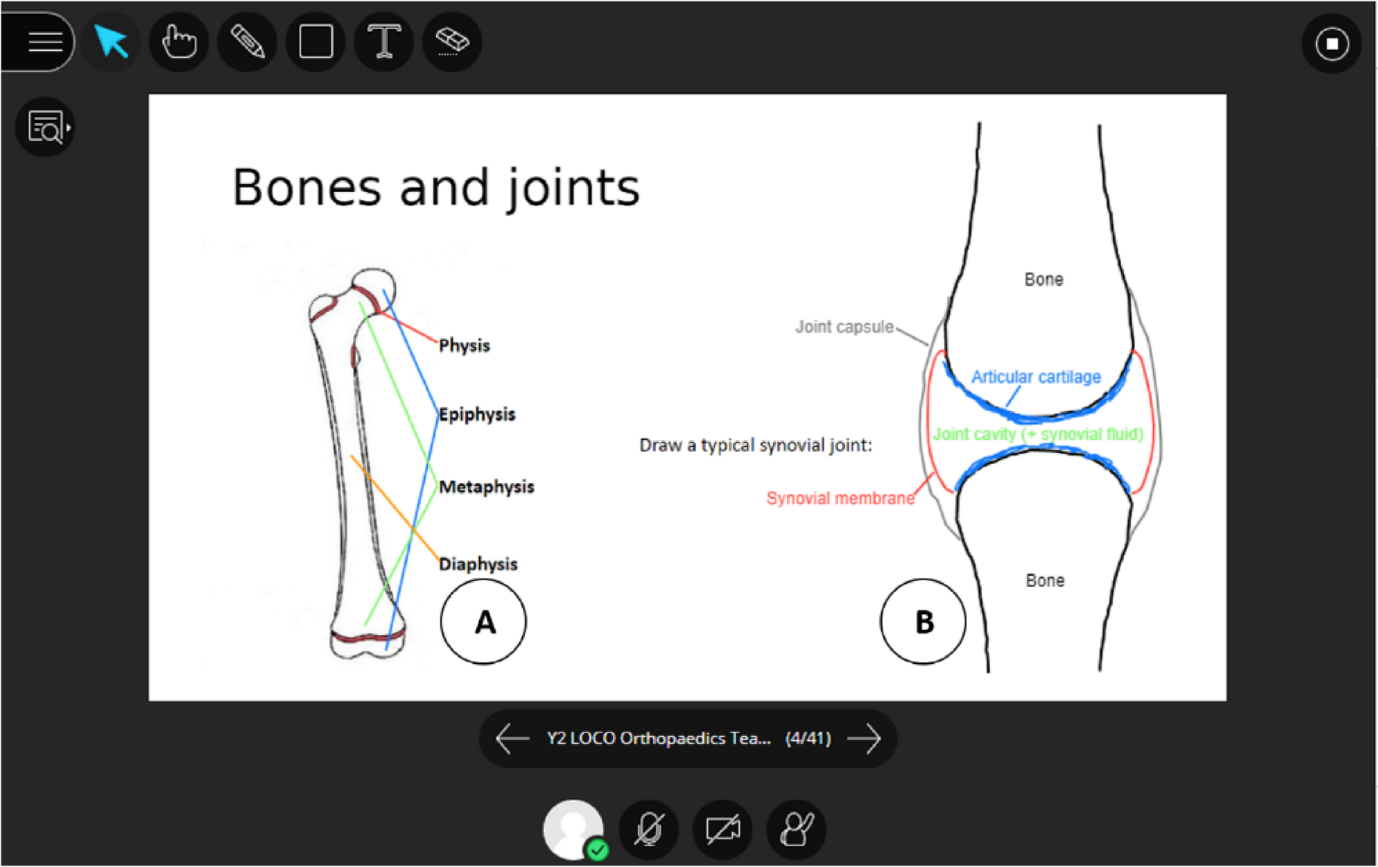
An example of learning activities involving annotation and drawing in BBC. Students were asked to draw lines between text and diagrams to label features (a) and draw (b) structures together [Color figure can be viewed at wileyonlinelibrary.com]

**FIGURE 3:**
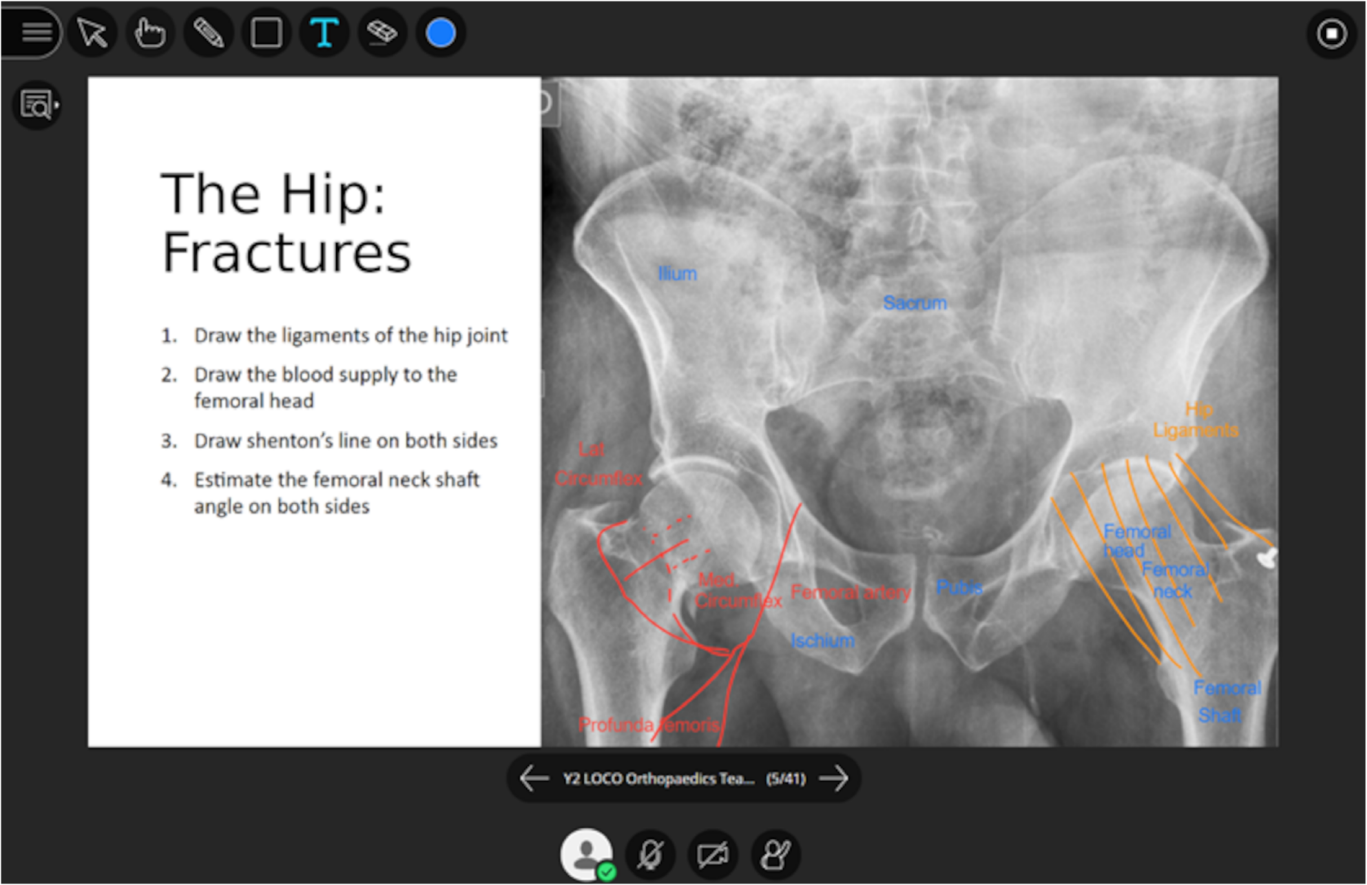
An example of collaboration in BBC. Students work together to label and interpret the positions of anatomical structures on a radiograph. BBC, Blackboard Collaborate [Color figure can be viewed at wileyonlinelibrary.com]

Most slides were designed to exploit the interactive and collaborative functions of BBC. PowerPoint files uploaded and internalized into the platform can be typed or drawn upon using a pencil tool, allowing custom slides to provide a canvas for the facilitation of a variety of learning activities. Annotations and drawings are instantaneously visible to all participants, and any number of students can perform these functions at once. We used these features to encourage students to work collaboratively in group activities (Figures 2 and 3). These included labeling key structures, questions to prompt discussion of key concepts, and challenging students to construct their own anatomical diagrams and models. Further interactive functions of BBC include an inbuilt polling system, through which facilitators can design custom multiple choice questions and answers to be presented during the session. This can be used to assess or gain feedback from students in real time. In our webinars, this was frequently made use of, with slides posing clinically related questions to test knowledge application, or to help tailor the agenda of the session to the preference of the group.

## 4 DISCUSSION

### 4.1 Faculty perspective

Over 2 months during lockdown in the UK, our department ran a total of 266 hr of synchronous online anatomy teaching. It was feasible between five educators to provide small group webinars to cover the learning objectives that would have been addressed in larger group F2F practicals. The faculty consensus was that most students engaged well in sessions, and sometimes better than generally occurs in F2F teaching. From our perspective, the use of 3D models and group learning activities during webinars were essential to their success. Incorporating these into the BBC platform helped us to maintain student engagement, synchronously test retention and application of knowledge, and use feedback to provide real‐time tailoring of the teaching agenda.

### 4.2 Building a virtual dissecting room

Learning anatomy involves developing an understanding of complex structures and their interactions with each other in space. Three dimensional modeling packages like Visible Body have emerged as a promising and effective way of learning anatomy outside the dissecting room (Nicholson, Chalk, Funnell, & Daniel, 2006). These programs allow exploration and manipulation of images to give the illusion of a 3D model on a computer screen, and improve learning in anatomy compared to 2D alternatives (Triepels et al., 2020; Yammine & Violato, 2015). More recently, with advances in video conferencing technologies, it has been possible to use modeling software to demonstrate 3D anatomy remotely with expert commentary. Attardi and Rogers (2015) were the first to describe this synchronously, using digital 3D models to create an online dissecting room. Students found these useful, and those taught online performed similarly in assessments to those taught in more traditional practicals (Attardi et al., 2018). We employed Visible Body in our webinars as it has been well rated for this purpose, although other programs are available (Attardi & Rogers, 2015; Lewis, Burnett, Tunstall, & Abrahams, 2014). We used virtual models in a similar manner to cadavers or plastic models in the physical dissecting room. This included demonstration and narration by teachers, but more importantly using them as stimuli to provoke student discussion, and as a reference to help students answer questions, including their own. Being able to refer to a 3D model in real time was invaluable to explain more difficult concepts. There are some potential benefits to using virtual models, other than remote availability. All students can gain a view and perspective identical to that of the teacher, and structures can be rapidly manipulated, dissected and zoomed in on, a feat not easily achieved in physical laboratories. Informal feedback suggested that students found the use of Visible Body 3D modeling software helpful, particularly when approaching more challenging subject matter.

Criticisms of a virtual dissecting room include a loss of emotional, olfactory, and tactile learning, as well as a limitation of students' experience of physiological and pathological anatomical variation (Longhurst et al., 2020). Although some students prefer less overwhelming virtual environments (Smith, Martinez‐Alvarez, & McHanwell, 2014), many educators feel that learning from cadaveric “patients” in the near clinical environment of the dissecting room is an essential part of anatomy education for future clinicians (Korf et al., 2008). Providing clinical context in anatomy teaching has been found to improve student performance (Bergman, Prince, Drukker, van der Vleuten, & Scherpbier, 2008). For many logistical reasons, including time, cost, and where legal limitations to the use of cadaveric images exist, this experience cannot be fully replicated online (Korf et al., 2008). Although the pathology and variation of cadaveric material may be missing, it is still possible to provide clinical context online. We situated anatomy problems within patient cases while incorporating clinical imaging, and referencing practical skills and procedures. Screensharing of recorded surgical procedures was not possible due to video lag, but may be more feasible on other platforms such as BigBlueButton.

### 4.3 Growing a virtual community

Fiock (2020) has recently set out practical guidance for how social, cognitive and teaching presences can be developed to grow a CoI. Based on our early experience, we have adapted their guidance for the purposes of online anatomy teaching, and provide a summary of recommendations in Table 1. Preliminary, informal feedback suggested that students found webinars useful, particularly valuing the small group nature, interactivity and ability to ask questions and work collaboratively on screen.

**TABLE 1:** Suggestions for developing social, cognitive and teaching presences in online anatomy teaching

Suggestion	Comments
*Social presence*	
Begin first sessions with video introductions of staff and students, with student orientation	Encourage 30 seconds of camera on for introduction; combine with collaborative activity for icebreaker and orientation to platform
Use preformed groups where possible	Students will often belong to an existing tutorial group
Aim for small group size, for example, between 8 and 16 students	Larger group sizes can fragment synchronous discourse online
Ensure all participants are visible and identifiable by name	If the platform does not provide names automatically, ask participants to write their name in the chat
Provide means for anonymous interaction	In BBC this is permitted by writing or drawing on slides
Encourage and model open discussion with sharing of experience, insight, and humor	Set the tone and register of the webinar, and model expectations for interaction by sharing experience
Design webinars which encourage group collaboration and discussion	Provide group tasks to stimulate interaction
Maintain the same group for the whole module	Timetable scheduled sessions and assign the same teacher for continuity
*Cognitive presence*	
Provide opportunity for problem solving and application of learning to a broader clinical context	Follow a practical inquiry model
Ground anatomical learning in real world experience	Make reference to clinical cases, cadaveric images and radiography
Provide opportunity for students to learn from each other and to lead group discussion	Delegate explanation of anatomical clinical problems to students
Encourage consideration of 3D relationship between structures in clinical context	Screenshare 3D anatomy modeling software to demonstrate key features
Provide opportunity for reflection on importance and relevance of anatomical structures	Set direct reflective activities or implicitly encourage discussion
Use a videoconferencing platform which allows synchronous activity and open discourse	Blackboard collaborate or other platforms may also be suitable
*Teaching presence*	
Teacher orientation prior to first session	Trial a session among staff prior to first webinar to familiarize with features of platform
Outline etiquette for online interaction	Provide rules of engagement for sessions in advance, for example, microphones are expected to be on/off
Design webinars which encourage active learning, critical thinking, and collaboration	Prompt reflection on the clinical relevance of particular anatomy
Allow students time to work through problems together	Online discussions can take longer than F2F discussions, resist the temptation to fill silences
Share own experience	Clinical staff may have relevant case examples from clinical practice
Seek regular feedback from students regarding understanding	Incorporate long pauses for audio feedback or invite the use of a polling system, or allow emoticons or faces drawn anonymously to display level of understanding
Be prepared to lead the session in the case of reduced student engagement	Teaching materials should be flexible to be used interactively or led by teacher. E.g. a drawing task can be used effective if student‐led or teacher‐led.
Offer a separate space where students can ask questions anonymously or in private, away from peers	This may be asynchronously via email or anonymous discussion board on the OLE, or synchronously by providing availability in a virtual office.

#### 4.3.1 Teaching presence

When designing online teaching, educators' decisions on the platform used and whether it will be synchronous or asynchronous will impact the development of a learning community. Asynchronous online discussion fora have been used frequently in anatomy but student engagement is often poor (De Leng, Dolmans, Muijtjens, & Van Der Vleuten, 2006; Green, Farchione, Hughes, & Chan, 2014; Swinnerton, Morris, Hotchkiss, & Pickering, 2017), with students complaining that these do not provide for sufficient levels of interaction in the absence of F2F teaching (Kelsey, McCulloch, Gillingwater, Findlater, & Paxton, 2020). We felt it was important to provide synchronous anatomy teaching, as immediacy in feedback can enhance communication, especially in circumstances where confusion is likely to occur (Steel & Jones, 2013). Providing structured opportunities for synchronous engagement to students has been shown to enhance learning experiences in blended learning courses (Stewart, Harlow, & DeBacco, 2011).

There seem to be an ever‐increasing number of online communication platforms available. We provide an overview of the current communicative and interactive functions of those that have been described for use in anatomy education in Table 2 (Attardi et al., 2018; Longhurst et al., 2020). Attardi et al. (2018) have previously shown that the communicative functions of BBC provide a virtual environment where students felt able to interact socially and academically with each other and staff. We sought to enhance a teaching presence further by enlisting the interactive functions of BBC that allow organization of group learning activities and frequent group polling. This allowed us to continually assess students' understanding, tailor teaching, and give individual feedback in real time, which is valued by students in distance learning environments (Martin, Parker, & Deale, 2012; Murphy & Rodríguez Manzanares, 2008).

**TABLE 2:** Communication and interactive functions of platforms used for synchronous anatomy teaching

Platform	Instant messaging chat bar	Allows anonymous questions	Polling function	Screen‐sharing	Teacher screen annotation	Student screen annotation
*Blackboard Collaborate (Blackboard Inc*.*)*	Yes	Yes	Yes	Yes	Certain programs only	Certain programs only
*Microsoft Teams (Microsoft)*	Yes	No	Yes	Yes	No	No
*Zoom (Zoom Video Communications*, *Inc*.*)*	Yes	No	Yes	Yes	Yes	No
*BigBlueButton (BigBlueButton Inc*.*)*	Yes	Yes	Yes	Yes	Certain programs only	Certain programs only

#### 4.3.2 Cognitive presence

To establish a cognitive presence, Garrison and Anderson (2003, p. 61) suggest following a practical inquiry model (PIM) composed of four phases (Table 3). We employed this model in sessions by creating a series of learning activities that incorporated clinical cases and imaging to provide a contextual basis for anatomical discussion. The collaborative functions of BBC were essential to allow the simultaneous exchange and integration of knowledge from multiple members of the group. As these interactions were often visually represented through drawing or annotating tools, it also provided a real‐time record of this collaboration (Figure 3).

**TABLE 3:** Practical Inquiry Model adapted from Garrison and Anderson (2003), p. 61)

Phase	Explanation	Example in BBC (Figure 3)
*Triggering the problem*	To instill a sense of puzzlement or dissonance	Presenting a clinical case, for example, a neck of femur fracture
*Exchanging information*	Exploring the issue by gathering and exchanging relevant information	Students contribute individual knowledge to the group through written or verbal communication. They record this exchange by editing slide content, for example, drawing the vasculature of the hip
*Integrating information*	Synthesis of information by connecting ideas in a meaningful way	Students connect the relationship between vascular compromise and fracture location
*Resolving the problem*	Solving the problem by applying and testing the ideas	Applying learning to interpret other cases of neck of femur fractures

Abbreviation: BBC, Blackboard Collaborate.

#### 4.3.3 Social presence

In addition to enabling synchronous communication, we felt it was important to provide flexibility in means of communication. Student preference for written or verbal communication varied significantly between groups. Students generally engaged well in discussion, and sometimes even better than we had previously experienced in F2F teaching. Many students engaged, often simultaneously, by using the chat bar and by writing or drawing on screen. The authors of screen annotations cannot be identified, and students also used this feature to ask questions anonymously that they might not otherwise have felt comfortable to ask in group teaching sessions. Some students feel more confident interacting in virtual as opposed to physical environments (McBrien, Jones, & Cheng, 2009; Murphy & Rodríguez Manzanares, 2008), and increased engagement for these students may be an advantage of moving teaching online.

One of the challenges in facilitating online teaching is a loss of non‐verbal communication (NVC) including body language, facial expression and eye contact. These provide a crucial source of instantaneous feedback on the contentedness and attention of students for teachers. Non‐verbal communication can be partially replaced through videoconferencing for very small groups (Nichol & Watson, 2000), but it is difficult to see how this might be achieved for larger classrooms, especially given the need to concurrently view or share teaching material. However, alternative methods of collecting similar information can be attempted. At review points during webinars, we asked students to doodle faces to represent how they felt about the topic that had been covered. This allowed teachers to virtually “read the room” for even large groups, gaining instantaneous, anonymized information about how many students were still engaged, and whether students wanted a new topic to be approached, or aspects of the current topic to be revised.

### 4.4 Adapting anatomy in context

It is important to consider the impact of transitioning to online anatomy teaching on the wider medical curriculum and student experience. This academic year a significantly greater proportion of the early medical curriculum will be delivered through remote means. There may be other important benefits to developing online learner communities outside of learning anatomy. It has been acknowledged that traditional F2F anatomy practicals serve as an important forum in facilitating early development of professional skills (Swick, 2006). These include communication, teamwork, and leadership skills that are grown through interaction between students and with staff (Flack & Nicholson, 2018; Ghosh, 2017). There is a need for anatomy education to continue to provide for development of these skills online, for use in the physical world but also for virtual professional contexts as online patient consultation becomes more commonplace (Sartori, Olsen, Weinshel, & Zabar, 2019). Facilitating collaborative, clinically relevant activities in anatomy webinars may help achieve this. Finally, reducing F2F elements of education has implications for student welfare. New and returning students will be more physically and socially isolated and there are concerns about the potential impact of this on their mental health and wellbeing (Gishen, Bennet, & Gill, 2020). Cultivating a sense of belonging and community through the anatomy curriculum may be one way to help mitigate this (Thomas, 2012).

## 5 CONCLUSION

At a time when social distancing measures are isolating students from family and friends, it may be more important than ever to provide a sense of community in our teaching. Although evidence and experience describing how best to do this in anatomy education is limited, distance‐learning pedagogy can inform the design of teaching to help develop new online communities of anatomical inquiry. We have found running synchronous teaching incorporating small group learning activities to be a viable approach, even for large student cohorts. Distance‐learning pedagogy would suggest that harnessing the collaborative and interactive functions now available in online communication platforms could develop social and cognitive presence in teaching. In our faculty's opinion these functions and the group learning activities they afforded were vital to maintain student engagement and enhance learning in webinars. The main limitations to our experience include that students and teachers were already known to each other from past F2F teaching, and that the evaluation occurred over 2 months only. Students who have not met peers or faculty members in a F2F setting, or who have more prolonged periods of online education may engage differently. Detailed investigation will be required to assess the impact of virtual curricula on anatomy education, and the student experience. Applying the Community of Inquiry framework may allow for an holistic evaluation of online anatomy education, and a survey instrument to facilitate this has been previously validated in healthcare education settings (Carlon et al., 2012). Future research could assess the validity of the CoI survey instrument in anatomy education, and compare the degree to which a CoI can be achieved across varying online approaches of different institutions.

## CONFLICT OF INTEREST

The authors declare no potential conflict of interest.

## AUTHOR CONTRIBUTIONS

William P. Flynn wrote the first draft of the manuscript. All authors reviewed and edited the manuscript and approved the final version of the manuscript.
